# Prospective assessment of breast lesions AI classification model based on ultrasound dynamic videos and ACR BI-RADS characteristics

**DOI:** 10.3389/fonc.2023.1274557

**Published:** 2023-11-03

**Authors:** Shunmin Qiu, Shuxin Zhuang, Bin Li, Jinhong Wang, Zhemin Zhuang

**Affiliations:** ^1^ Department of Ultrasound, First Affiliated Hospital of Shantou University Medical College, Shantou, Guangdong, China; ^2^ School of Biomedical Engineering, Sun Yat-sen University, Shenzhen, Guangdong, China; ^3^ Product Development Department, Shantou Institute of Ultrasonic Instruments, Shantou, Guangdong, China; ^4^ Department of Ultrasound, Shantou Chaonan Minsheng Hospital, Shantou, Guangdong, China; ^5^ Engineering College, Shantou University, Shantou, Guangdong, China

**Keywords:** artificial intelligence, diagnosis, ultrasound video, BI-RADS, breast

## Abstract

**Introduction:**

AI-assisted ultrasound diagnosis is considered a fast and accurate new method that can reduce the subjective and experience-dependent nature of handheld ultrasound. In order to meet clinical diagnostic needs better, we first proposed a breast lesions AI classification model based on ultrasound dynamic videos and ACR BI-RADS characteristics (hereafter, Auto BI-RADS). In this study, we prospectively verify its performance.

**Methods:**

In this study, the model development was based on retrospective data including 480 ultrasound dynamic videos equivalent to 18122 static images of pathologically proven breast lesions from 420 patients. A total of 292 breast lesions ultrasound dynamic videos from the internal and external hospital were prospectively tested by Auto BI-RADS. The performance of Auto BI-RADS was compared with both experienced and junior radiologists using the DeLong method, Kappa test, and McNemar test.

**Results:**

The Auto BI-RADS achieved an accuracy, sensitivity, and specificity of 0.87, 0.93, and 0.81, respectively. The consistency of the BI-RADS category between Auto BI-RADS and the experienced group (Kappa:0.82) was higher than that of the juniors (Kappa:0.60). The consistency rates between Auto BI-RADS and the experienced group were higher than those between Auto BI-RADS and the junior group for shape (93% vs. 80%; *P* = .01), orientation (90% vs. 84%; *P* = .02), margin (84% vs. 71%; *P* = .01), echo pattern (69% vs. 56%; *P* = .001) and posterior features (76% vs. 71%; *P* = .0046), While the difference of calcification was not significantly different.

**Discussion:**

In this study, we aimed to prospectively verify a novel AI tool based on ultrasound dynamic videos and ACR BI-RADS characteristics. The prospective assessment suggested that the AI tool not only meets the clinical needs better but also reaches the diagnostic efficiency of experienced radiologists.

## 1 Introduction

According to the latest statistics on cancer incidence and mortality from the International Agency for Research on Cancer (GRIBOCAN) in 2020, breast cancer incidence has risen to the top and become the first cause of death among women worldwide (1). Early screening for breast cancer is crucial to reducing death rates. National guidelines for breast cancer screening vary from country to country. Due to the high proportion of dense breasts in Chinese women and the low sensitivity of mammography, the National Cancer Centre of China proposes that the ultrasound (US) should be the preferred method for breast cancer screening in Chinese women and recommends that women over 45 years old should be screened by ultrasound alone every 1-2 years (2). China has a large population which brings the heavy workload of breast cancer ultrasound screening. Therefore, it is necessary to develop a clinical application AI tool that can assist in diagnosis quickly and efficiently.

In clinical practice, to improve the accuracy of diagnosis, standardize ultrasound description, and communicate effectively with the physician, worldwide radiologists generally use the American College of Radiology Breast Imaging Reporting and Data System (ACR BI-RADS) lexicon for breast US (3). The radiologist scans the whole breast with handheld ultrasound and gives BI-RADS category. However, since handheld ultrasound depends on the operators and experience, different radiologists have different opinions on the interpretation of BI-RADS characteristics, resulting in a high inter-observer variability, poor repeatability and low work efficiency (4–6).

To the best of our knowledge, AI is the most likely tool to improve diagnostic effectiveness and reduce the subjective and experience-dependent nature of handheld ultrasound. In recent years, with the continuous application of AI in clinics, deep learning has been favored by human experts due to its strong capacity for autonomous feature extraction and expression (7). Several studies applied deep learning to classify US images of breast lesions and have reported that it could achieve a high diagnostic performance similar to or better than that of experienced radiologists. Becker et al. (8) used a deep neural network to identify malignant lesions in 637 breast lesions. Han et al. (9) used the GoogLeNet convolutional neural network to classify benign and malignant ultrasound images of 7408 breast lesions. However, these studies are all based on a keyframe image which was not in accord with the actual situation of the clinical ultrasound dynamic scan. Moreover, a single static image cannot contain all the information about the entire breast lesion. In addition, studies (10, 11) showed that one person may also have other diagnoses within videos and static images for one lesion. Youk et al. (12) showed that in radiologists’ interpretation of BI-RADS characteristics, videos had a higher diagnostic performance than static images. What’s more, the above studies all belong to the benign and malignant dichotomy, which is of little clinical guiding significance compared with the multi-classification of BI-RADS. Ciritsis et al. (13) and Qian et al. (14) tried to use deep learning to conduct multi-classification studies of BI-RADS on breast lesions. However, they all merged BI-RADS 4a, 4b, and 4c into category 4, which was not in line with clinical practice, and at the same time, they still failed to overcome the limitations of using static images.

To overcome the above limitations, we first proposed an approach to scan the breast lesions and record their ultrasound dynamic videos per unified criteria, obtaining ACR BI-RADS morphological characteristics, and realizing the BI-RADS category. Compared to traditional methods based on single-frame static images, it not only captures comprehensive and complete breast lesion information, avoiding missing lesion features in static images but also better suits clinical diagnostic scenarios. In this approach, we introduce an AI diagnostic model (hereafter, Auto BI-RADS), which includes a YOLOV5 network with improved attention mechanism and morphological image processing algorithms. Based on effectively screening, localizing, and capturing tumor lesions in breast ultrasound dynamic videos, Auto BI-RADS can obtain BI-RADS morphological characteristics, achieve BI-RADS category and make a benign or malignant prediction. In this study, we prospectively verified its performance through a comparative test.

## 2 Materials and methods

### 2.1 Study sample

**Figure 1:**
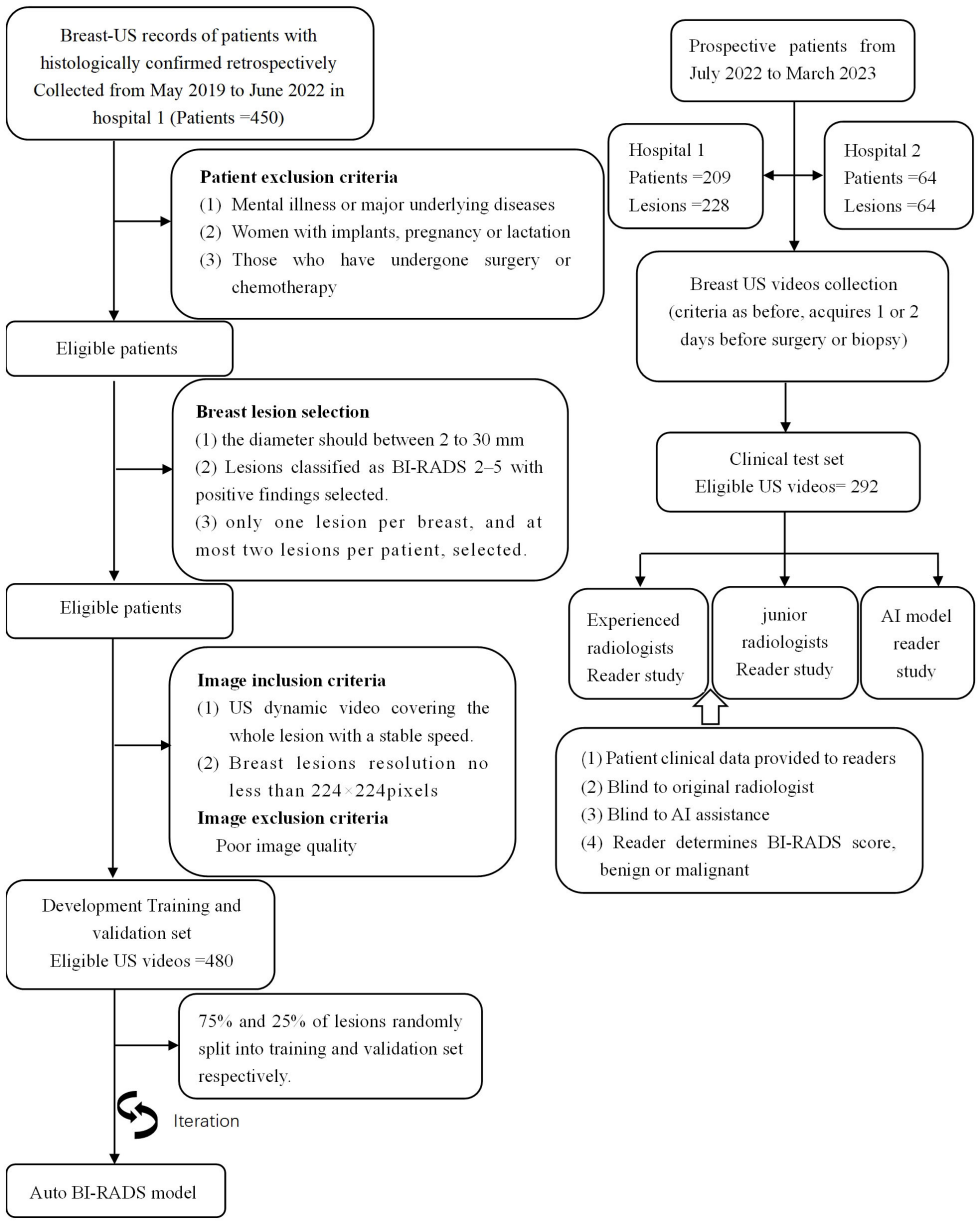
Overview of the retrospective and prospective workflow. It should be pointed out that all BI-RADS categories in this study were determined on 2D US videos exclusively.

The institutional review board approved this study, and the requirement to obtain informed consent was waived (approval number: B-2022-182). In the development of Auto BI-RADS, we include retrospective data from the First Affiliated Hospital of Shantou University Medical College (Guangdong, China) with a total of 480 pathologically proven lesions. **Figure 1** presents the inclusion and exclusion criteria. A total of 480 ultrasound dynamic videos equivalent to 18122 static images comprised the training and validation sets at 3:1 (mean age, 45 years; range, 18–82 years, May 2019 to June 2022). In the testing study, the dataset was screened with the same criteria and included two hospitals: internal test set (mean age 45 years, range,19-76 years, First Ultrasound Department, First Affiliated Hospital of Shantou University Medical College[Hospital 1], July 2022 to March 2023, n = 228); and external test set (Mean age: 50 years old; range,26-73 years old; Ultrasound Department, Shantou Chaonan Mingsheng Hospital [Hospital 2], July 2022 to March 2023, n = 64). A flowchart describing the research process is shown in **Figure 1**. Baseline clinical pathologic data, including age, sex, pathologic findings, and US diagnosis reports, were derived from the medical records. US video data were recorded by two experienced radiologists per the criteria below (SM.Q., with 7-8 years of experience, Hospital 1; JH.W., with 11-12 years of experience, Hospital 2).

### 2.2 US examination

US examinations were performed with linear array transducers of real-time US systems. All patients in hospital 1 were examined with the following US scanners: Canon Toshiba (Japan, Aplio I800, L9-18 MHz), SIUI (China, Apogee 6800, L8-12 MHz), and Siemens (Germany, Acuson S3000, L9-12MHz). Patients in hospital 2 were examined with Siemens (Germany, Acuson Sequoia, L4-10MHz).

US video acquisition: The patients held the supine position and raised their hands to fully expose their axilla and breast. We selected the maximum transverse diameter section of breast lesions and used the body mark showing the position; adjusted the depth to place the lesions in the center of the screen and the focus at the bottom of them; activated the storage function; kept the transducer at a constant speed to scan the lesions until some normal breast tissues appear, and pressed the storage key to acquire the video.

### 2.3 US image analysis

**Figure 2:**
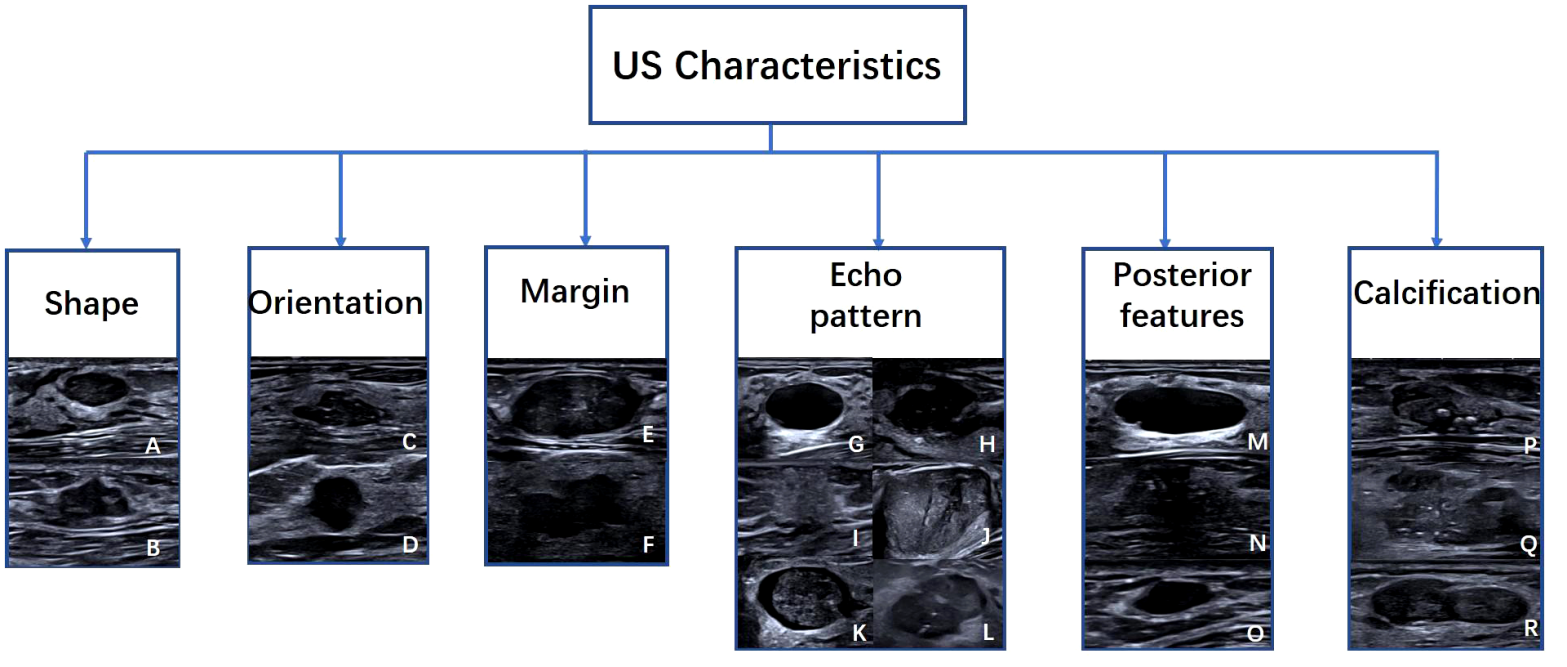
Examples of US images with Six American College of Radiology Breast Imaging Reporting and Data System characteristics. Characteristics include **(A)** regular, **(B)** irregular, **(C)** parallel, **(D)** not parallel, **(E)** circumscribed, **(F)** not circumscribed, **(G)** Anechoic, **(H)** hypoechoic, **(I)** hyperechoic, **(J)** Isoechoic, **(K)** complex cystic and solid, **(L)** heterogeneous, **(M)** enhancement, **(N)** shadowing, **(O)** no posterior features, **(P)** macrocalcifications, **(Q)** punctate echogenic foci, **(R)** no calcification.

The six lexicon categories of BI-RADS were labeled as identifying features (shape, orientation, margin, echo pattern, posterior acoustic features, and calcification). The shape was a binary classification feature: regular or irregular; orientation was also binary: parallel or not parallel; the margin was another binary feature: circumscribed or not circumscribed. The echo pattern was mapped to three binary classification features: Anechoic, homogeneous echo (including homogeneous low, equal, and high echoes), heterogeneous echo (including heterogeneous solid and cystic-solid echoes); Posterior acoustic features are also classified into three classification features: no change, enhancement, or shadowing. Calcification is the last category, classified into three classification features: no calcification, coarse calcification, and punctate calcification (**Figure 2**).

In the training set, two experienced radiologists (DP.L. and XX.C, both with 10 years of US experience) were blinded to histopathologic results and independently manually labeled masks for each breast lesion video. Image classification was then performed based on the fifteen features of the six main BI-RADS lexicon. Groups would make a discussion to reach a consensus. In the validation dataset, the initial performance of Auto BI-RADS was evaluated by those two radiologists.

For the test data set, four radiologists blinded to histopathologic results were split into two groups: experienced radiologists (BQ.Z. and XY.L., with 30 and 28 years of ultrasound work experience, respectively) and junior radiologists (ZY.L. and X.C., with 3 and 4 years of ultrasound work experience, respectively). Each radiologist independently evaluated the features of breast lesions in dynamic videos and determined the benign or malignant nature of the mass. When the evaluation results were inconsistent, a group consensus was reached through discussion.

### 2.4 AI model development

#### 2.4.1 The establishment of Auto BI-RADS diagnosis model

**Figure 3:**
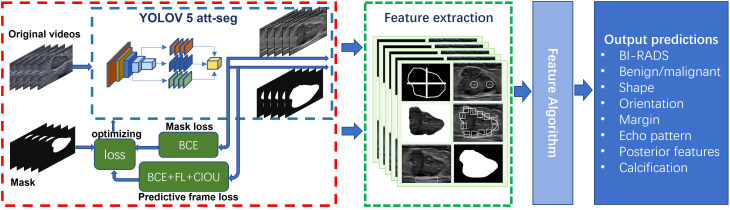
Proposed network scheme of the Auto BI-RADS model for breast lesions diagnosis based on American College of Radiology Breast Imaging Reporting and Data System US characteristics.

The diagnostic AI model of Auto BI-RADS consists of three parts (**Figure 3**). The first part is based on the YOLOV 5 attention and segment network for object detection and segmentation. This model first converts the input breast lesions US video into sequence frames and selects frames containing lesions, then extracts and segments the regions of interest and their corresponding masks (15–18). In order to improve the detection performance of the network, we added the simple attention mechanism (Sim AM) to the model, which enhances the recognition effect of the small breast tumor target. We also combined the binary cross entropy loss (BCE loss) function, focal loss (FL loss) function, and Complete-Intersection-Over-Union (CIOU) loss function to optimize the network (19, 20). The second part focuses on extracting features of breast lesions using image processing algorithms. In this part, tumor regions of interest and their corresponding masks are obtained. These regions of interest and masks undergo equalization processing and data augmentation (21). After that, morphological image processing algorithms are used to extract features such as shape, orientation, margin, echo pattern, posterior acoustic features, and calcification from these tumor slices. The third part involves a feature score fusion algorithm based on weighted thresholds. Because multiple and unevenly distributed features may present in the breast lesions ultrasound video sequences, we establish threshold values based on the proportion of frames in which different features appear. Then, we merge the scores of all detected features and use a rank threshold score table to divide the tumor into its BI-RADS category and distinguish its benign or malignant nature.

**Figure 4:**
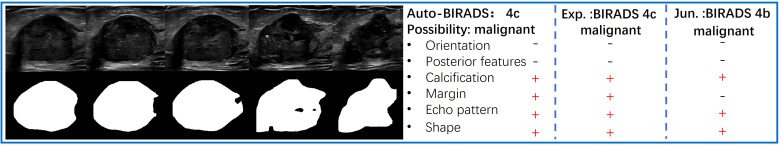
The left side displays a series of key frames extracted from an ultrasound dynamic video of a breast lesion that was pathologically proven as malignant. The right side shows the predictive results of the Auto BI-RADS model for the lesion based on ACR BI-RADS, as well as the interpretation results of the experienced (Exp.) and junior (Jun.) radiologists.

The predictive results of the Auto BI-RADS include the BI-RADS category, benign or malignant, and the assessment results of six significant features for each nodule. **Figure 4** shows the prediction results of the Auto BI-RADS model in a case, compared with the interpretation results of experienced and junior radiologists.

#### 2.4.2 The design of the YOLOV5 att-seg network based on the Sim AM

The deep learning model in this study is the YOLOV5 network based on the Sim AM. Hereafter we call it the YOLOV5 Attention Segmentation model (YOLOV5 att-seg).

**Figure 5:**
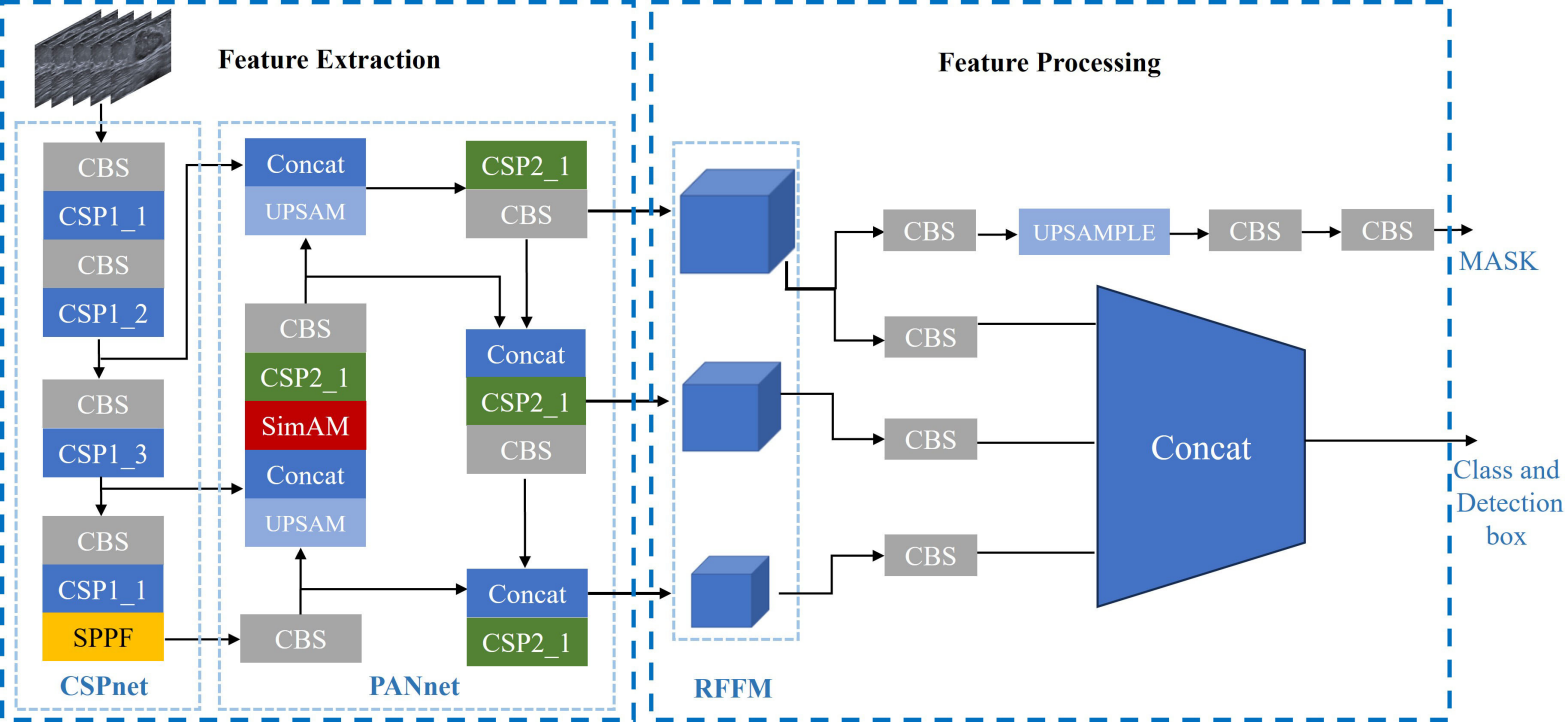
YOLOV5 att-seg Architecture.

The YOLOV5 network model consists of feature extraction and feature processing. The feature extraction part includes a Cross Stage Partial Network (CSP Net) (21) and a Path Aggregation Network (PA Net) (16) (**Figure 5**).

The CSP net is primarily composed of multiple Conv+BatchNorm+SiLU (CBS) modules, Cross Stage Partial modules 1(CSP1), and spatial pyramid pooling fast (SPPF) modules. This network extensively utilizes residual structures and convolutional modules for refining image features and reducing feature map dimensions through downsampling. Additionally, it preserves feature maps at different depths within the network, allowing subsequent parts of the PA Net network to further integrate features from different levels.

**Figure 6:**
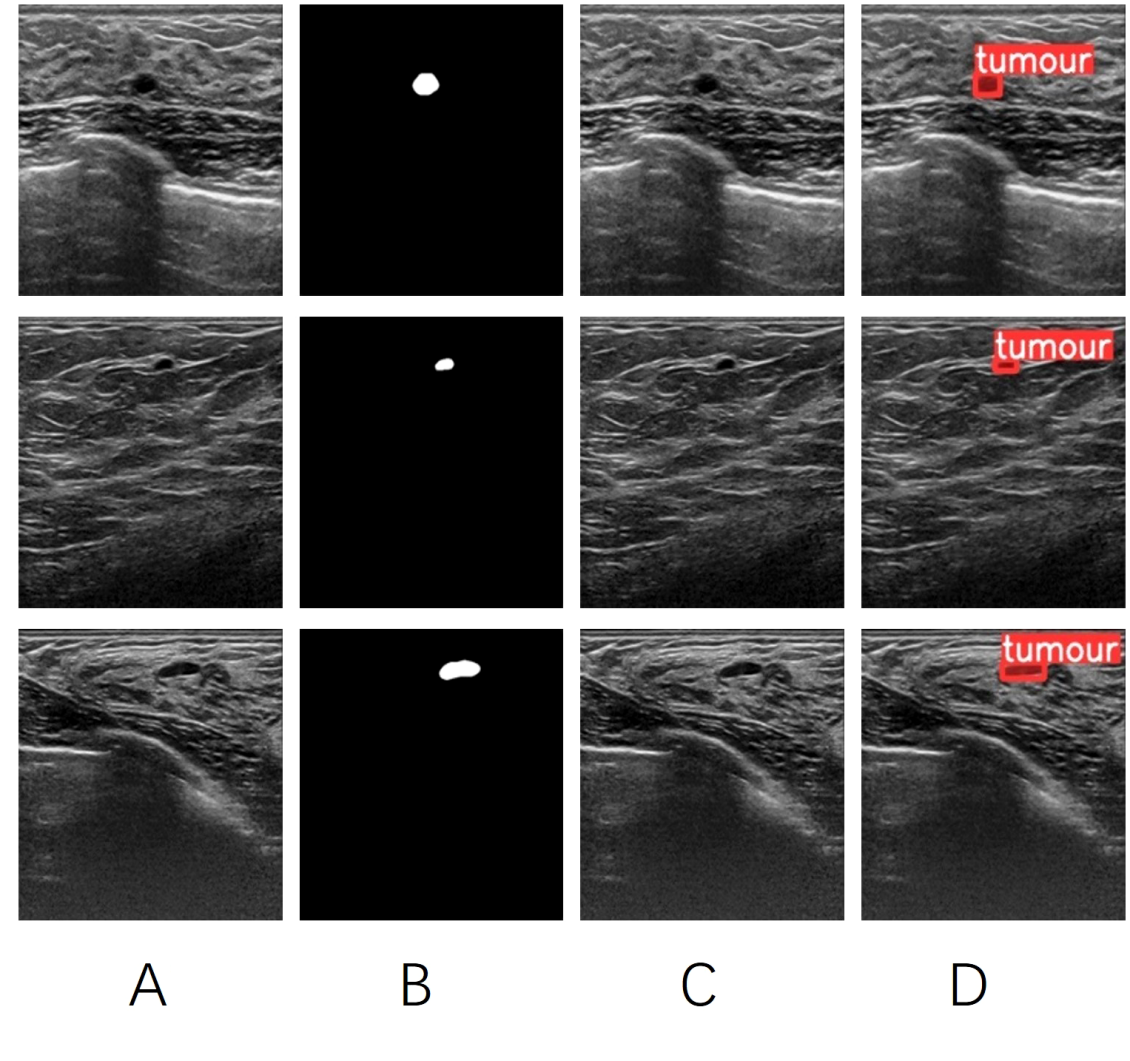
**(A)** the original image of a breast tumor. **(B)** the manually annotated ground truth by experienced physicians. **(C)** the detection results of YOLOV5 seg. **(D)** the detection results of YOLOV5 att-seg.

The PA Net Network is primarily used for generating feature pyramids to enhance the model’s detection of objects at different scales. It is an improvement based on the Feature Pyramid Network (FPN) architecture. The network consists of CBS modules, CSP2 modules, and Sim AM modules. Since CSP Net already captures sufficiently deep-level feature information, a non-residual module called CSP2 is used in the PA Net section to accelerate training and inference speed. The inclusion of Sim AM aims to further enhance the network’s detection performance for small lesions. Sim AM is a parameter-free attention mechanism module based on the theory of neural energy functions (17). It calculates the neural energy of the input image and performs Hadamard multiplication with the input image to spontaneously enhance or suppress the neural pathways. (**Figure 6**) shows that YOLOV5 without Sim AM failed to identify tumor targets in some small breast tumor slices. However, the YOLOV5 att-seg model, which incorporates the Sim AM, exhibited improved detection performance for small tumor targets.

The second part is the feature processing section. It mainly involves processing the feature information obtained from the feature extraction section. The previous CSP Net and PA Net have effectively refined and aggregated the image features. Therefore, this part of the work is divided into two branches: mask segmentation and tumor target detection. One branch utilizes upsampling layers and CBS modules to further refine the edge information of the features, thereby obtaining better details of the mask edges. The other takes the three different-sized receptive field feature maps (RFFM) output by the PA Net network, adjusts them to the same dimension using CBS modules, and fuses them with the Concat function to enhance the network’s detection of objects of different sizes. Finally, the network outputs the categories and detection boxes (22).

#### 2.4.3 Performance validation of the YOLOV5 att-seg network model

**Figure 7:**
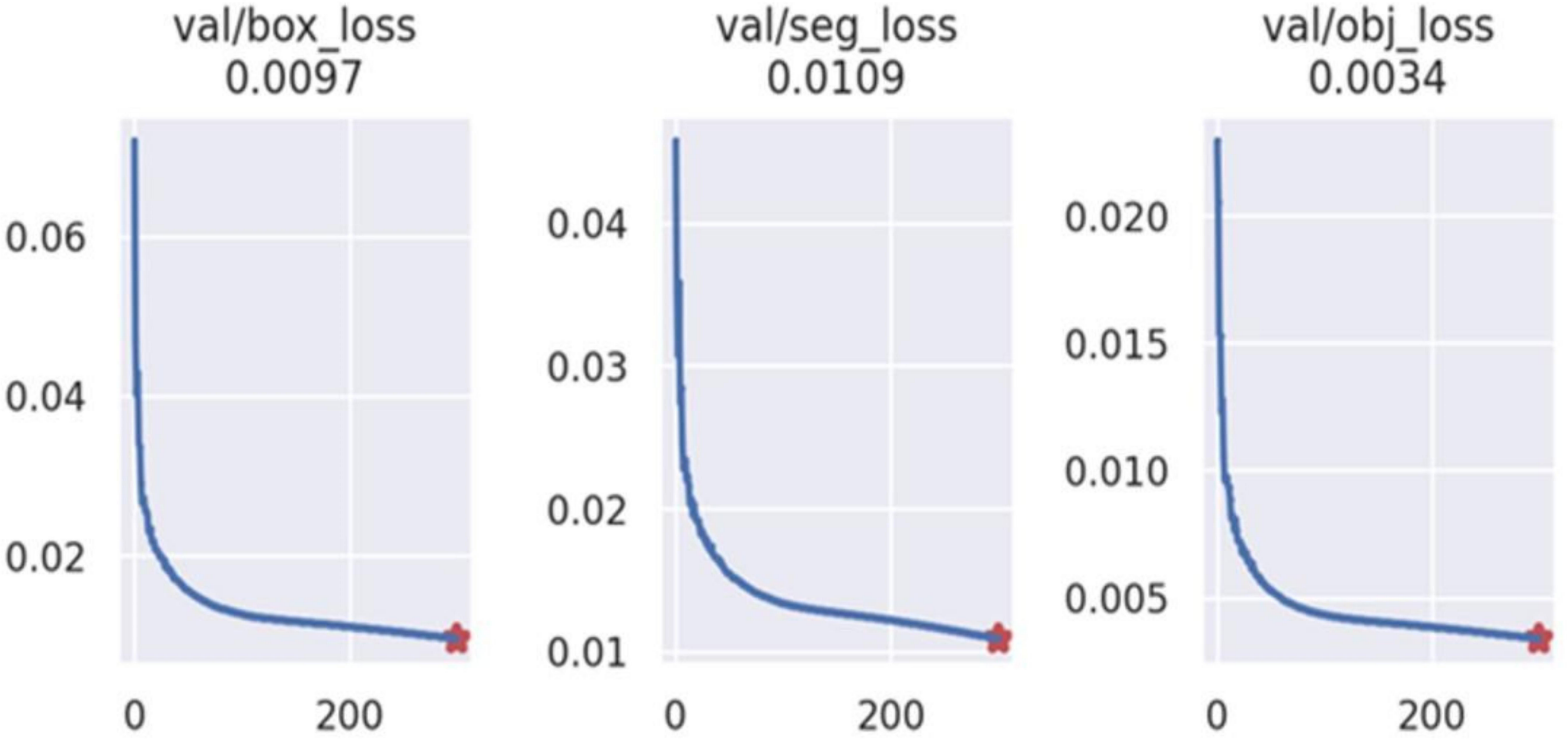
The box loss, segmentation loss, and target category loss of YOLOV5 att-seg achieve 0.0097, 0.0109, and 0.0034, respectively. It indicates that the network has achieved a good fit to the overall data.

The YOLOV5 att-seg network in this study was trained using 18122 static images extracted from 480 ultrasound dynamic videos. The detection box data was obtained by extracting the bounding rectangles from the masks. 75% of the samples were used for network training. The network was trained for 300 epochs, and the results are shown in (**Figure 7**).

In the remaining 25% samples for validation, we compared the detection performance of YOLOV5 att-seg with YOLOV5, Vgg-16 and Resnet50 networks. We also compared the segmentation performance of YOLOV5 att-seg with YOLOV5, Unet and Fcn-16s networks. The result is shown in **Table 1**.

**Table 1:** Comparison of performance metrics for object detection and segmentation in validation set.

Network	Function	Precision	Recall	Specificity	Dice.	Iou.
YOLOV5 att-seg	det./seg.	0.98|0.98	0.93|0.93	0.94|0.94	0.77	0.68
YOLOV5	det./seg.	0.97|0.98	0.92|0.93	0.91|0.91	0.75	0.67
Vgg-16	det.	0.84	0.86	0.82	–	–
Resnet50	det.	0.78	0.77	0.77	–	–
Unet	seg.	0.60	0.76	0.97	0.62	0.53
Fcn-16s	seg.	0.53	0.66	0.96	0.53	0.46

det, detection; seg, segmentation; Dice, Dice coefficient; Iou, Intersection over Union.

The detection result shows that YOLOV5 att-seg has an improved performance in detecting smaller tumors in ultrasound images. In comparison, YOLOV5 att-seg vs. YOLOV5 vs. Vgg-16 vs. Resnet50, the precision, recall, and specificity are (0.98, 0.93, 0.94, vs. 0.97, 0.92, 0.9, vs. 0.84, 0.86, 0.82, vs. 0.78, 0.77, 0.77), respectively.

The segmentation result shows that YOLOV5 att-seg has an improved performance in precision, recall, Dice. and Iou. comparing with YOLOV5, Unet and Fcn-16s (0.98, 0.93, 0.77,0.68, vs. 0.98, 0.93, 0.75,0.67, vs. 0.60, 0.76, 0.62,0.53, vs. 0.53, 0.66, 0.53,0.46, respectively). However, the specificity of YOLOV5 att-seg (0.94) was slightly lower than that of Unet (0.97) and Fcn-16s (0.96). This indicates that the YOLOV5 att-seg model used in this study achieves a more balanced performance compared to Unet and Fcn-16s. Unet and Fcn-16s tend to have overly conservative segmentation contours for ultrasound tumor targets, resulting in abnormally high specificity values. On the other hand, the enhancement in small target detection of YOLOV5 att-seg leads to improved segmentation performance compared to YOLOV5.

In terms of network running speed, YOLOV5 att-seg achieves significant improvement by using a single network to extract image features and obtain detection boxes and segmentation masks for tumor targets. This network demonstrates much faster speed compared to the traditional approach using separate networks for detection and segmentation.

### 2.5 Statistical analysis

The areas under the receiver operating characteristic curves (AUCs) with 95% confidence intervals (CI) were compared using the DeLong test (23) for Auto BI-RADS and two groups of radiologists. The threshold of Auto BI-RADS was established using validation sets. Performance metrics (sensitivity, specificity, positive predictive value, and negative predictive value) of Auto BI-RADS and the two groups of radiologists were evaluated. The Kappa test was used to compare the consistency of the breast lesion BI-RADS category between Auto BI-RADS and the two groups of radiologists. The McNemar test was used to compare the consistency rate of breast lesion characteristics recognition among Auto BI-RADS, the experienced group, and the junior group. Data were analyzed with SPSS, version 26.0 (IBM), and MedCalc, version 20.2 (MedCalc Software). *P*<0.05 was considered indicative of a statistically significant difference.

## 3 Results

### 3.1 Patient characteristics and clinical features of breast lesions

A total of 698 patients were included in this study. In model development, 420 patients (480 pathologically confirmed lesions: 284[60%] benign and 196 [40%)] malignant) from Hospital 1 were collected for training and validation in a 3:1 ratio. In the test data set, there were 278 patients (292 pathologically confirmed lesions: 168 [58%] benign and 124 [42%] malignant) from Hospitals 1 and 2. **Figure 2** shows the workflow of patient inclusion and exclusion for model development and independent test. The specific pathological composition and distribution of the lesions are shown in **Table 2**.

**Table 2:** Patient demographics data and breast lesion characteristics.

Characteristic	Retrospective Training and Validation sets	Prospective test sets	
		Hospital 1 Set	Hospital 2 Set
Number of patients	420	214	64
Age (years) (mean)	45 ± 12	45 ± 12	50 ± 11
Number of lesions	480	228	64
Lesion maximum diameter(mm)			
2-10	167(35%)	89(39%)	13(20%)
10-20	185(38%)	90(40%)	34(53%)
20-30	128(27%)	49(21%)	17(27%)
BI-RADS category ^a^			
2	34(7%)	18(8%)	0(0%)
3	110(23%)	30(13%)	10(16%)
4a	106(23%)	70(31%)	8(13%)
4b	80(17%)	41(18%)	14(22%)
4c	74(15%)	35(15%)	22(34%)
5	76(16%)	34(15%)	10(15%)
Lesion type			
Invasive ductal carcinoma	82(17%)	26(11%)	14(22%)
Invasive lobular carcinoma	73(15%)	43(19%)	16(25%)
Ductal carcinoma *in situ*	21(4%)	10(4%)	6(9%)
Other malignant ^b^	20(4%)	4(2%)	5(8%)
Fibroadenoma	141(30%)	64(28%)	13(20%)
Other benign ^c^	143(30%)	81(36%)	10(16%)

The BI-RADS category ^a^ is based on the interpretation of the radiologist who originally performed the US examinations before the biopsy test, not the radiologists involved in the reader study. It should be noted that all BI-RADS categories involved in this study were determined on breast-US video images only. ^b^ Includes non-specific malignant results. ^c^ Includes adenosis, hyperplasia, benign phyllodes tumors, and papillomata.

### 3.2 Performance of Auto BI-RADS experienced radiologists and junior radiologists for diagnosing benign and malignant breast lesions

**Figure 8:**
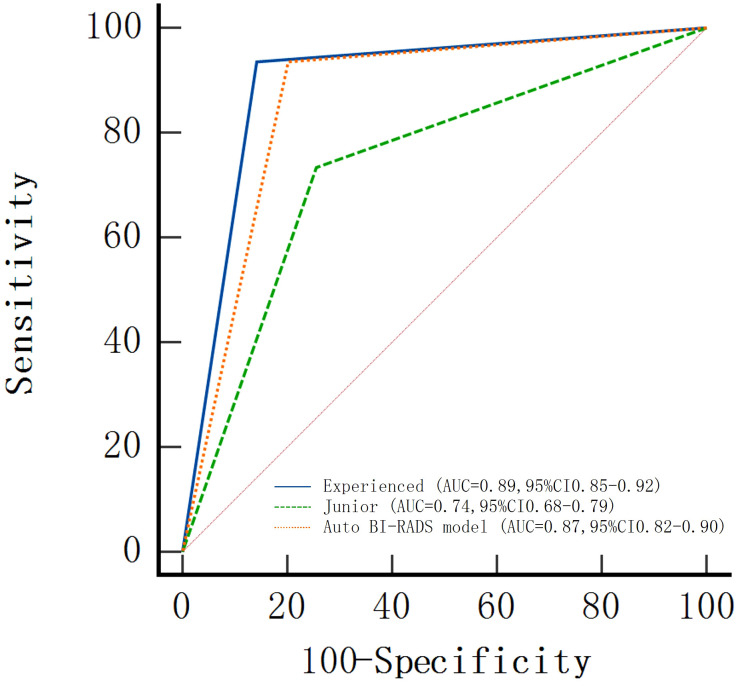
Areas under the receiver operating characteristic curves (AUCs) of the Auto BI-RADS model for breast lesions based on the American College of Radiology Breast Imaging Reporting and Data System and two radiologist groups with different experience levels who used BI-RADS.

In the test set, the AUC value was slightly lower than that of the experienced group but significantly higher than that of the junior group as shown in **Figure 8**.

The AUC, sensitivity, specificity, positive predictive value, and negative predictive value of Auto BI-RADS were 0.87 (95%CI: 0.82, 0.90), 93% (116 out of 124 lesions), 81% (136 out of 168 lesions), 78% (116 out of 148 lesions), and 94% (136 out of 144 lesions), respectively. The false positive of the Auto BI-RADS is 6%, as much as the experienced group. We found no evidence of a statistical difference between the Auto BI-RADS model and the experienced group for AUC (*P* = 0.06), but there were statistically significant differences compared to the junior group (*P* < 0.001) (**Table 3**).

**Table 3:** Performance of Auto BI-RADS and two groups of radiologists for diagnosis of benign and malignant breast lesions in test set.

Parameter	Auto BI-RADS	Experienced Radiologists	Junior Radiologists
AUC	0.87[0.82,0.90]	0.89[0.85,0.92]	0.74 [0.68,0.79] ^†^
Sensitivity (%)	93(116/124)	93(116/124)	72 (91/124)
Specificity (%)	81(136/168)	86(144/168)	74(125/168)
FP (%)	19(32/168)	14(24/168)	26(43/168)
FN (%)	6(8/124)	6(8/124)	25(33/124)
PPV (%)	78(116/148)	82(116/140)	68(91/134)
NPV (%)	94(136/144)	95(144/152)	79(125/158)

—Except where indicated, numbers in parentheses are numbers of lesions and 95% confidence intervals are in brackets.

FP, False Positive; FN, False Positive.

NPV, negative predictive value; PPV, positive predictive value.

† Data are for comparison with Auto BI-RADS (P < 0.001).

### 3.3 Comparison of consistency among Auto BI-RADS, experienced radiologists, and junior radiologists in the BI-RADS category in the test set

In the test set of 292 breast lesions, the consistency between the Auto BI-RADS model and the experienced radiologists in the BI-RADS category was higher than that of the junior radiologists, with kappa values of 0.82 and 0.60, respectively (**Table 4**).

**Table 4:** Consistency rates between Auto BI-RADS model and two radiologist groups for classification of BI-RADS in test set.

US Characteristic	Rate between Auto BI-RADS and Experienced Radiologists (%)	Kappa Value	Rate between Auto BI-RADS and Junior Radiologists (%)	Kappa Value
BI-RADS 2	100(17/17)	0.82	82(14/17)	0.60
BI-RADS 3	97(36/37)		51(21/37)	
BI-RADS 4a	84(68/81)		77(63/81)	
BI-RADS 4b	80(42/52)		53(28/52)	
BI-RADS 4c	83(50/60)		70(42/60)	
BI-RADS 5	88(40/45)		75(34/45)	

-Numbers in parentheses are numbers of lesions (n = 292).

Cohen’s Kappa coefficient is used to a diagnostic method. A Kappa value of 0.4–0.59 indicated weak agreement, 0.6–0.79 indicated moderate agreement, 0.8–0.9 indicated strong agreement and values above 0.9 indicated perfect agreement between two references.

### 3.4 Comparison of consistency rates among Auto BI-RADS, experienced radiologists, and junior radiologists in the identification of breast lesions characteristics in the test set

In the test set of 292 lesions, the consistency rate between Auto BI-RADS and experienced radiologists was higher than that between Auto BI-RADS and junior radiologists in the identification of morphology, orientation, margin, internal echo, posterior echo with respective values for morphology (93% [n = 271] vs. 80% [n = 234]; *P* = 0.01), orientation (90% [n = 265] vs. 84% [n = 247]; *P* = 0.02), margin (84% [n = 246] vs. 71% [n = 209]; *P* = 0.01), internal echo (69% [n = 202] vs. 56% [n = 163]; *P* = 0.01) and posterior echo (76% [n = 221] vs. 71% [n = 207]; *P* = 0.046). In the identification of calcification, there was no statistically significant difference in the consistency rates between Auto BI-RADS and experienced radiologists or junior radiologists (*P* = 0.4) (**Table 5**).

**Table 5:** Consistency rates between Auto BI-RADS Model and two radiologist groups for identification of breast lesions characteristics in test set.

US Characteristic	Rate between Auto BI-RADS and Experienced Radiologists (%)	Rate between Auto BI-RADS and Junior Radiologists (%)	*P* Value
Shape	93(271)	80(234)	0.01
Orientation	90(265)	84(247)	0.02
Margin	84(246)	71(209)	0.01
Echo pattern	69(202)	56(163)	0.01
Posterior features	76(221)	71(207)	0.046
Calcification	31(91)	29(85)	0.4

-Numbers in parentheses are numbers of lesions (n = 292).

## 4 Discussion

In this study, we first developed a breast lesions AI classification model. By identifying the BI-RADS characteristics within the ultrasound dynamic videos, it can automatically evaluate the lesions’ BI-RADS category and predict their benign or malignant nature.

The development of the Auto BI-RADS model was based on 480 breast lesions ultrasound videos equivalent to 18122 static images from Hospital 1, with a 3:1 ratio for training and validation. To verify the stability and efficiency of the model, we made an independent test in this study in 292 breast lesions testing sets from Hospital 1 and Hospital 2. Compared with those of experienced and junior radiologists, it showed that Auto BI-RADS achieved high performance in distinguishing between benign and malignant breast lesions (AUC: 0.87, sensitivity: 93%, specificity: 0.81), which was close to the experienced radiologists (AUC: 0.89, sensitivity: 93%, specificity: 86%), and significantly better than juniors (AUC: 0.74, sensitivity: 72%, specificity: 74%). Tracing back to the previous studies, Han et al. (9) first used an end-to-end deep learning framework to classify regions of interest selected by radiologists in a dataset of 7,408 static ultrasound breast lesions. They reported a sensitivity of 0.86, specificity of 0.93, and AUC >0.9. Ciritsis et al. (13) used a deep learning model that mimicked human decision-making to detect and classify ultrasound breast lesions in a dataset of 1,019 static images. In an external test dataset, they reported a sensitivity of 0.894, specificity of 1.0, and AUC of 0.967. Qian et al. (14) developed a neural network model that combined ultrasound B-mode and color Doppler to classify static ultrasound images of the breast in a larger dataset. Their bimodal model reported an AUC of 0.982, specificity of 88.7%, and sensitivity of 97%. Although the diagnostic performance indicators reported in those studies may appear higher than our study, they are not directly comparable. Firstly, the above studies were based on keyframes that can reflect the main BI-RADS characteristics of breast lesions. However, in clinical practice, not all radiologists were able to select the most critical frames. Secondly, a single static image cannot reflect all the morphological features of one breast lesion. Therefore, the results of those studies may have significant bias and low reproducibility, with limited clinical applicability. Additionally, none of the above studies conducted independent testing, raising questions about the stability of the models. In contrast, we used ultrasound dynamic videos for independent testing, which could improve its clinical generalizability with more objective and reproducible consequences.

In addition, we also compared the consistency among Auto BI-RADS, experienced radiologists, and junior radiologists in the BI-RADS category (the Kappa values were 0.82 and 0.60, respectively). The results showed that Auto BI-RADS were highly consistent with experienced radiologists. Finally, we compared the identification of breast lesions’ BI-RADS characteristics. The results showed that Auto BI-RADS had a higher consistency rate with experienced radiologists in morphology, orientation, margin, internal echo, and posterior echo. This indicates that the model conforms to the visual judgment of experienced human experts. These comparative studies have not been mentioned in previous studies. As for the recognition of calcification, there was no difference among the Auto BI-RADS model, experienced and junior radiologists. We speculate that this is because the characteristics of calcification are more complex, and their distribution in terms of location, size, and shape varies greatly. The image algorithm identifies different grayscale thresholds to determine whether calcification exists, and it will fail when there are only show slight changes in grayscale. It was also found that some tumors with high echogenic envelopes were mistakenly identified as calcifications. Chen et al. (24) had similar explanations in their identification of thyroid calcification. Later, we will increase the calcification samples and improve the algorithm to enhance the identification of calcification.

To meet the practical application, we first developed the Auto BI-RADS model based on ultrasound dynamic videos combining deep learning and image processing algorithms. There have been no similar reports previously. In the first step, we employed a YOLOV5 deep convolutional neural network to track and segment the targets. Then, we utilized image processing algorithms to extract BI-RADS features. Finally, we performed feature algorithm fusion to obtain target classification. For video tracking and segmentation, Yap et al. (25) have compared multiple types of deep learning neural networks, demonstrating their powerful capabilities in object tracking and segmentation. Relevant studies (26–28) have also indicated that deep learning exhibits uncertainty and a lack of interpretability in lesion feature recognition. Continuous learning required large sample sizes for the identification of each specific feature (29). However, machine learning has unique advantages in extracting breast lesion features. Hamyoo et al. (30) used machine learning alone to extract 13 features from lesions using the BI-RADS lexicon in a multi-center study (1288 static ultrasound images from three countries: Malaysia, Iran, and Turkey) and obtained an AUC value of 0.88, demonstrating the strong feature recognition capabilities of machine learning through comparison with human expert readings. Herein, our study constructed an Auto BI-RADS model based on deep learning and image processing algorithms to achieve the identification and classification of breast lesions in ultrasound dynamic videos. The prospective assessment indicates that the Auto BI-RADS model demonstrates good diagnostic performance and has significant potential.

Reviewing other imaging for breast cancer screening, mammography is considered a recommended method for reducing breast cancer-related mortality, but it involves radiation and is less sensitive to dense breasts, making it unsuitable for all countries (31). MRI is always used as a supplementary means (31). On the other hand, the US is recommended by Asia medical experts due to its low cost, non-radiation, and suitability for Asian women (31). The limitations of ultrasound have always been operator dependence and observer variability. Although many studies have focused on developing artificial intelligence models to address these limitations, they have not fully taken into account the practical clinical applications. Considering the above problems, we propose to develop a novel AI model simulating the clinical practice conducted by dynamic videos and BI-RADS characteristic identification. This approach allows for more objective, realistic, and reliable diagnostic results with high repeatability. The application of Auto BI-RADS offers great practical significance and provides better references for clinical practitioners with less experience.

Our study has several limitations. Firstly, the sample size for the prospective evaluation of the model is not large enough, and it does not include all categories of BIRADS, especially category 1 which indicates no lesion. Afterward, to improve the adaptability and stability of the model, we will include more external hospitals to increase the samples and species. By strengthening the model’s training, we increase the model’s robustness. Furthermore, it should be noted that the breast lesions ultrasound videos used in this study may still exhibit variability due to handheld ultrasound. In the future, if an Automated Breast Ultrasound System (ABUS) or robotic arms can be used to record the videos, it would provide more convincing results. Additionally, the latest fifth edition ACR BI-RADS guidelines have added color Doppler and elastography to evaluate breast lesions (32). It means that multimodal ultrasound has become part of breast cancer assessment. Therefore, for further improvement, this study can incorporate multiple ultrasound modalities such as color Doppler, elastography, contrast-enhanced ultrasound, etc., to develop a multimodal AI ultrasound diagnostic model.

## 5 Conclusion

In conclusion, we first propose a novel method for breast tumor AI diagnosis based on breast lesions ultrasound dynamic videos to obtain ACR BI-RADS morphological characteristics, realize the BI-RADS category, and predict benign or malignant lesions. In the AI model development, we combined an improved attention mechanism YOLOV5 network with image processing algorithms to achieve it. This novel method not only avoids the problem of missing and incomplete lesion features caused by traditional single-frame static images but also better suits clinical diagnostic scenarios, providing a fast and effective approach for breast cancer screening.

## Data availability statement

The original contributions presented in the study are included in the article/supplementary material. Further inquiries can be directed to the corresponding authors.

## Ethics statement

The studies involving humans were approved by the Institutional Review Board of First Affiliated Hospital of Shantou University Medical College(approval number: B-2022-182). The studies were conducted in accordance with the local legislation and institutional requirements. The ethics committee/institutional review board waived the requirement of written informed consent for participation from the participants or the participants’ legal guardians/next of kin because the data are anonymous.

## Author contributions

ZZ: Conceptualization, Funding acquisition, Methodology, Resources, Supervision, Writing – original draft, Writing – review & editing. SQ: Conceptualization, Data curation, Formal Analysis, Investigation, Methodology, Resources, Writing – original draft. SZ: Methodology, Project administration, Software, Validation, Visualization, Writing – review & editing. BL: Investigation, Software, Validation, Writing – review & editing. JW: Data curation, Formal Analysis, Investigation, Writing – review & editing.
